# Impact of multicomponent exercise and nutritional supplement interventions for improving physical frailty in community-dwelling older adults: a systematic review and meta-analysis

**DOI:** 10.1186/s12877-024-05551-8

**Published:** 2024-11-18

**Authors:** Wachiranun Sirikul, Nida Buawangpong, Kanokporn Pinyopornpanish, Penprapa Siviroj

**Affiliations:** 1 Department of Community Medicine, Faculty of Medicine Chiang, Mai University, Chiang Mai, 50200 Thailand; 2 Department of Family Medicine, Faculty of Medicine Chiang, Mai University, Chiang Mai, 50200 Thailand; 3 Center of Data Analytics and Knowledge Synthesis for Health Care, Chiang Mai University, Chiang Mai, 50200 Thailand; 4 Environmental and Occupational Medicine Excellence Center, Chiang Mai University, Chiang Mai, 50200 Thailand; 5 Department of Biomedical Informatics and Clinical Epidemiology, Faculty of Medicine, Chiang Mai University, Chiang Mai, 50200, Thailand

**Keywords:** Frailty, Older adults, Multicomponent exercise, Nutritional interventions

## Abstract

**Objective:**

To investigate the efficacy of both multicomponent exercise and nutritional interventions on frailty by conducting a systematic review and meta-analysis to examine changes in frailty incidence.

**Design:**

A systematic review and meta-analysis.

**Eligible criteria:**

The included studies were limited to original controlled trials focused on frailty interventions in older adults aged 65 years and over. The studies involved only participants with specific diseases, and those recovering from surgery or being hospitalized were excluded.

**Information sources:**

A systematic search was performed on three databases: PUBMED, EMBASE, and Cumulative Index to Nursing and Allied Health, with the latest search in October 2024. Three authors independently extracted the data using a standardized data collection form. Relative risks were used as a summary measure. Pooled-effect estimates of each outcome were calculated by the random-effects meta-analysis.

**Results:**

After searching three databases, 5327 records were identified. After removing duplicates and screening the titles and abstracts, 19 multicomponent exercise studies and 7 nutritional intervention studies were eligible. In a pooled analysis of 18 multicomponent exercise RCTs, including a total of 3457 older adults, the multicomponent exercises showed a clinically significant reduction in frailty risk by relative change 55% times (95% CI 45% to 67%, *p* value < 0.001). The subgroup analysis of combinations of macronutrients and micronutrients also demonstrated statistically significant decrease in frailty risk by relative change 28% times (95% CI 11% to 72%, *p* value = 0.008).

**Conclusion:**

Multicomponent exercises can effectively improve physical frailty, regardless of the duration and types of the activities, whereas the efficacy of nutritional supplements remains unclear. Personalized multicomponent approaches that incorporate both exercises and nutritional supplements have promised to enhance effectiveness in reducing frailty, thus warranting further investigation.

**Trial registration:**

The study was registered on 12 September 2022, under PROSPERO registration number CRD42022357357.

**Supplementary Information:**

The online version contains supplementary material available at 10.1186/s12877-024-05551-8.

## Question

What are specific interventions and standard suggestions for the duration and intensity of the interventions for frailty?

## Finding

From 18 multicomponent exercise studies and 6 nutritional intervention studies, the multicomponent exercises showed a clinically significant reduction in frailty risk by relative change 55% times (95% CI 45% to 67%, *p* value <0.001). The subgroup analysis of combinations of macronutrients and micronutrients also demonstrated statistically significant decrease in frailty risk by relative change 28% times (95% CI 11% to 72%, *p* value = 0.008).

## Meaning

Multicomponent exercises can effectively improve physical frailty, regardless of the duration and types of the activities, whereas the efficacy of nutritional supplements remains unclear. Multicomponent approaches that incorporate both exercises and nutritional supplements have promised to enhance effectiveness in reducing frailty, thus warranting further investigation.

## Introduction

As the world growing in the aging population, frailty is common syndrome that occur in 5% to 7% of older people [1]. Frailty refers to a condition characterized by increased vulnerability and diminished physiological reserve in individuals, often associated with the aging process. Frailty patients often experience dysfunction in multiple organ systems. This is due to age-related factors, including a decline in muscle mass and an increase in inflammation, which can significantly impact their overall health [2]. There are various derived concepts of frailty, such as physical frailty [3], cognitive frailty [4], and social frailty [5], and various evaluation methods have been proposed.

To identify individuals with physical frailty, various screening tools are employed. One common tool is Fried's Frailty Phenotype [6], which assesses weight loss history, feelings of fatigue (measured using the CES-D Depression Scale), physical activity levels (evaluated with the Modified Global Physical Activity Questionnaire), walking time, and grip strength. Physical frailty can significantly impact an individual's quality of life and independence. Individuals who are frail have a higher susceptibility to adverse health outcomes, including falls, disability, hospitalization, and mortality [7, 8]. Therefore, the attempt to reverse back to prefrail or healthy status in frailty patient is currently being studied [9].

Medical professionals can assess frailty in older adults to identify individuals at higher risk and implement appropriate interventions. Engaging in regular exercise, maintaining a balanced diet, and participating in social activities are key factors in preventing or delaying the onset of frailty [10]. There are also various studied methods that can improve frailty [3, 11]. Management strategies for frailty may include tailored exercise programs [12], nutritional support [13], underlying medical conditions and medication reviews [14], and fall prevention [15].

Although, previous research often indicates a decrease in the frailty index or physical performance with these interventions [16, 17]. There is also limited meaningful objective evidence on the incidence of frailty following specific interventions and standard suggestions for the duration and intensity of the interventions [18, 19]. Isolated nutritional intervention may not be effective for the management of frailty [20]. These interventions need a crucial and in-depth study. Moreover, the authors were aware of the previous meta-analyses on physical activity, multicomponent interventions, and nutritional interventions for improving physical frailty prior to conducting this study. The estimates presented in these investigations were standard mean differences in frailty scores rather than incidences [21–24]. A substantial decrease in frailty scores does not directly indicate that frailty has been converted to robustness. The need for a result refinement process further highlights the clinical benefit of these interventions for frailty treatment and prevention. Therefore, this systematic review and meta-analysis encompasses randomized controlled trials (RCTs) and controlled clinical trials (CCTs) from the bibliographic databases with the aim of examining the efficacy of multicomponent exercise and nutritional interventions in reducing the incidence of frailty.

## Materials and methods

### Searching strategies and information sources

Initially, systematic searches of three major bibliographic databases, including PUBMED, EMBASE, and Cumulative Index to Nursing and Allied Health (CINAHL), were performed in September 2022. The most recent search was conducted in October 2024. In order to find previously unidentified references, we performed forward and backward citation searches on Google Scholar. We also contacted the corresponding authors of the eligible studies if the full-text article or some important information was not readily available. In this study, we focused on a study involved the community-dwelling older adults aged 65 and over. The interventions of interest were an multicomponent exercise training and a nutritional intervention that aim to decrease or maintain the outcome of interest, physical frailty status in accordance with Fried’s definition or Cardiovascular Health Study (CHS) frailty phenotypes. The keywords related to exercise and nutritional interventions (including exercise, physical activity, and nutrition), physical frailty (frailty and debility), and older adults, were combined together to construct a comprehensive search term for retrieving all relevant studies. The details of the search strategy for each database and search results were described in the supplementary material “Search strategy”. This study was carried out in accordance with the systematic literature review and meta-analysis reporting guidelines of the Preferred Reporting Items for Systematic Reviews and Meta-Analysis (PRISMA) 2020 [25], and it was registered on 12 September 2022, under PROSPERO registration number CRD42022357357.

### Eligibility criteria

The retrieved studies were selected based on the following criteria: (1) all types of intervention studies included randomized controlled trials (RCTs), controlled clinical trials (CCTs), and controlled before and after studies; (2) an original English-language article published from year 2000 onwards; (3) the exercise interventions provided in the following settings and modes of delivery were included: home-based and center-based (e.g., workplace, community and day centers, nursing home, and primary care) interventions; public, private, voluntary, or commercial interventions; and interventions delivered by healthcare professionals, home caregivers, laypeople or volunteers, researchers, the media, and the Internet; (4) the nutritional interventions included macronutrient dietary supplements (e.g., protein, carbohydrate, and fat), micronutrients (e.g., minerals, vitamins, phenolic compounds, total flavonoids, and anthocyanin), or both; (5) reported outcome on the effectiveness of exercise or nutritional interventions in the community-dwelling older adults aged 65 years and over for reducing or preventing physical frailty status as defined by Frailty phenotypes [6]. The exclusion criteria were as follows: (1) a study involved only participants with the specific diseases (e.g., cancer, stroke, fragility fracture) or those recovering from surgery or being hospitalized; (2) review articles or letters to the editor; (3) articles with only an abstract presented at the conference.

### Data collection process and data items

Three researchers (W.S., P.S., and N.B.) independently assessed the titles and abstracts of the initial 100 records and debated discrepancies until consensus was reached. Two independent reviewers (W.S. and P.S.) then independently screened the titles and abstracts of all retrieved articles in pairs. In the event of disagreement, screening of full-text articles was decided through discussion. The third reviewer (N.B.) was consulted if necessary to make the final judgment. Next, two independent reviewers (W.S. and P.S.) independently evaluated full-text articles for inclusion and carried out the data collection process using the standardized record form. The extracted data included information on (1) the study: authors, publication date, country, study design, and study base (center-based, home-base, or both); (2) the participants: baseline characteristics (age, gender, and frailty status) and the number of participants in each study arm; (3) the exercise interventions: modes of exercise (strength, balance, flexibility, and endurance), mode of delivery, duration per session, frequency, length of intervention, and the number of drop-out participants. The multicomponent exercise program was defined as a combination of endurance, strength, coordination, balance, and flexibility exercises [26, 27], following the recommendations of the American College of Sports Medicine [28]; (4) the nutritional interventions: the type of nutrition (macronutrients, micronutrients, or both), dose and frequency, length of intervention, and the number of drop-out participants; (5) the comparator: the type of assignment (e.g., placebo, usual care, health education, alternative interventions, or no intervention.); and (6) the outcomes: the number of frail and non-frail participants in each study arm and the duration of follow-up. If there were unclear issues or missing data in the studies that were included, disagreements were solved by the consensus of the reviewers and consultation with the third reviewer (N.B.).

### Study risk of *bias* and reporting *bias* assessment

According to the included studies' research designs, the risk of bias was evaluated. For RCTs, Version 2.0 of the Cochrane risk-of-bias tool for randomized trials (RoB2.0) was used to evaluate the risk of bias related to the randomization process, interventions, missing outcome data, and selection of the reported results [29]. The risk of bias in each domain was rated ranging from "low risk of bias" to "some concerns" to "high risk of bias" and the overall risk of bias judgement for each study will be made in accordance with the criteria of RoB2.0. For CCTs or Non-RCTs, Risk Of Bias In Non-randomized Studies—of Interventions (ROBIN-I 2016) was used to evaluate the risk of bias due to confounding, selection of participants, interventions, missing outcome data, measurement of outcomes, and selection of the reported results [30]. The risk of bias in each domain and the overall risk of bias judgement was rated as either "low risk of bias", "moderate risk of bias", "serious risk of bias", and "critical risk of bias".

Each study’s risk of bias was evaluated independently by two reviewers (P.S. and W.S.). For the purpose of assessing potential bias resulting from selective publication and selective reporting of results, we will also retrieve study protocols and trial registrations if they are available. The third reviewer (N.B.) will be contacted if disagreements cannot be resolved through discussion between the two reviewers (W.S. and P.S.). The results of RoB2.0 and ROBIN-I assessment were presented as a traffic light plot and a summary plot using Robvis tool [31].

### Effect measures and synthesis methods

The effect estimates of this study were reported as the pooled relative risk of physical frailty between the intervention and control groups and were visualized by a forest plot. The information of the eligible studies, including the details of participants characteristics, study designs, intervention, control, and outcome measurements, are presented in tabular format. Cochran Q and I^2^ tests were used to confirm the significant and degree of heterogeneity of the eligible studies. I^2^ value were interpreted the degree of heterogeneity as follows: low heterogeneity (I^2^ < 40%); moderate heterogeneity (I^2^ 30–60%); substantial heterogeneity (I^2^ 50–90%); and considerable heterogeneity (I^2^ 75–100%) [32]. A meta-analysis of the eligible studies was performed using Restricted Maximum Likelihood (REML) random-effects model for estimating the pooled effect estimates if these studies are sufficiently homogeneous (I^2^ < 60%) or if heterogeneity is sufficiently diminished by the analysis in pre-specified subgroups, including length of the multicomponent exercise interventions (three, six and more than twelve months) and type of the nutritional interventions (macronutrients, micronutrients, or both).

Evaluation of the possible bias from small-study effects (e.g., publication bias) was examined through funnel plot visualization. Regression-based tests, Egger's and Harbord test, were applied to identify any potential publication bias in a meta-analysis with ten or more included studies. If asymmetry in the funnel plot or statistical significance (*p* < 0.050) of tests If asymmetry were found, we intended to examine the study characteristics to determine whether it was likely caused by publication bias or by other characteristics like methodological or clinical heterogeneity in the studies. Sensitivity analysis was performed to evaluate the robustness of effect estimates and assess the causes of heterogeneity from the primary analysis. Sensitivity analyses on the impact of study designs (RCT/non-RCT), interventions (types of intervention, lengths of intervention), and exclusion of small studies or high risk of bias studies from meta-analysis as well as the impact of fixed-effect (the inverse-variance method) or random-effects models on summary measures were performed. Furthermore, a comprehensive narrative synthesis was performed, and any relevant and important characteristics of the eligible studies will be qualitatively described, especially for a meta-analysis result with a high degree of heterogeneity. A sensitivity analysis of small-study effects by trim and fill method was additionally performed if there was evidence of reporting bias from asymmetry of funnel plot or statistical tests for reporting bias. All statistical analyses and visualizations for a meta-analysis were conducted using STATA17 (Stata Corp. 2019, Stata Statistical Software: Release 17, Stata Corp LLC, College Station, TX, USA). Finally, the certainty of evidence for the meta-analysis outcomes was evaluated by the two reviewers (W.S. and P.S.) using the GRADE approach [33].

## Results

### Study selection and characteristics

**Fig. 1:**
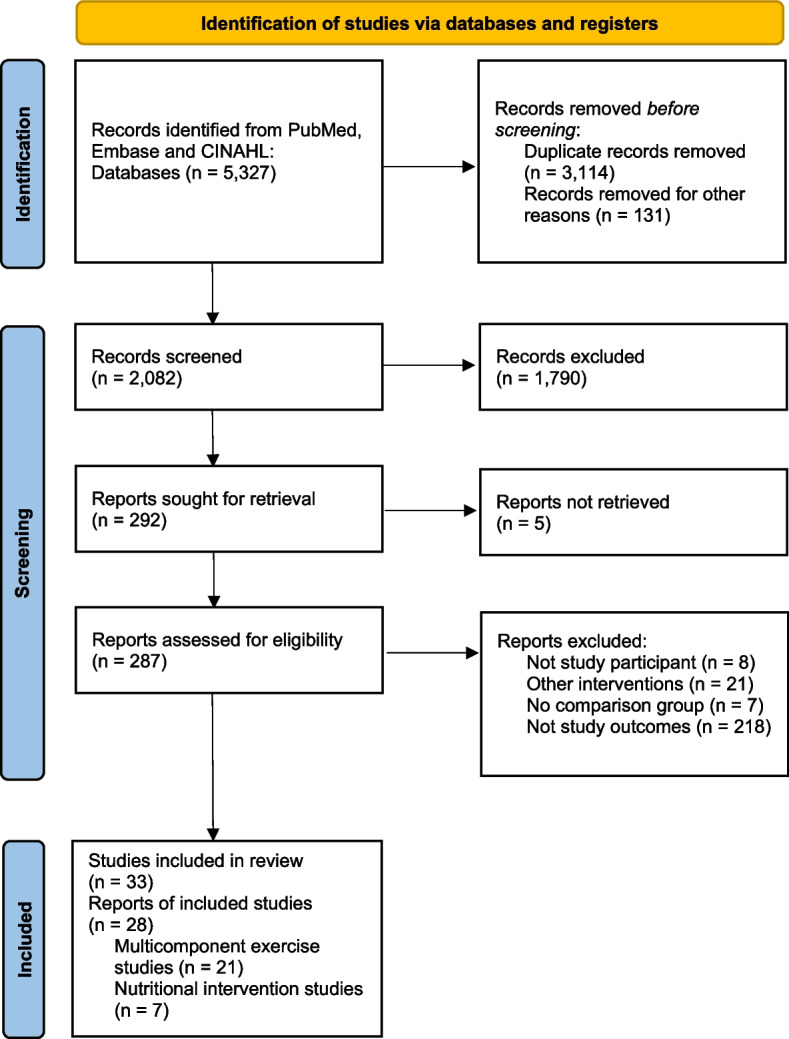
PRISMA flow diagram is placed here

As shown in the PRISMA flow diagram (Fig. 1), we identified 4,106 records from searching the three databases during initial search (19 September 2022). After removing duplicates, we screened the titles and abstracts of 2,082 records, from which we reviewed 33 full-text articles and included 21 multicomponent exercise studies and seven nutritional intervention studies. After full-text review, five multicomponent exercise studies, including three CCTs (Moreira, N.B. et al., 2020 [34]; Merchant, R.A. et al., 2021 [35]; and Santos de Abreu Garcia, R.N. et al., 2021 [36]) and two RCTs (Costa, S.N. et al., 2020 [37]; and Yu, R. et al., 2019 [38]), were excluded from a systematic review and meta-analysis. Moreira, N.B. et al., 2020 [34], were excluded due to no reported frailty incident cases. Santos de Abreu Garcia, R.N. et al., 2021 [36], and Merchant, R.A. et al., 2021 [35], compared different exercise programs, but there was no non-exercise control group. Costa, S.N. et al., 2020 [37] was excluded due to a high risk of bias in multiple domains via the RoB 2.0 assessment. Yu, R., et al. 2019 [38] were also excluded because of the absence of a non-exercise control group and the use of combined interventions (computerized cognitive training plus physical exercises). Additionally, we searched for articles that cited any of the initially included studies, as well as the studies' references. However, no further articles that fulfilled eligible criteria were identified during these searches. Finally, the included studies on multicomponent exercises were 17 RCTs and two CCTs. The included studies on nutritional intervention were six RCTs and one CCT. The characteristics of the included studies for full-text reviews are shown in Supplementary Tables S1 for the multicomponent exercise and Supplementary Tables S2 for the nutritional intervention studies.

### Risk of *bias* in studies

**Fig. 2:**
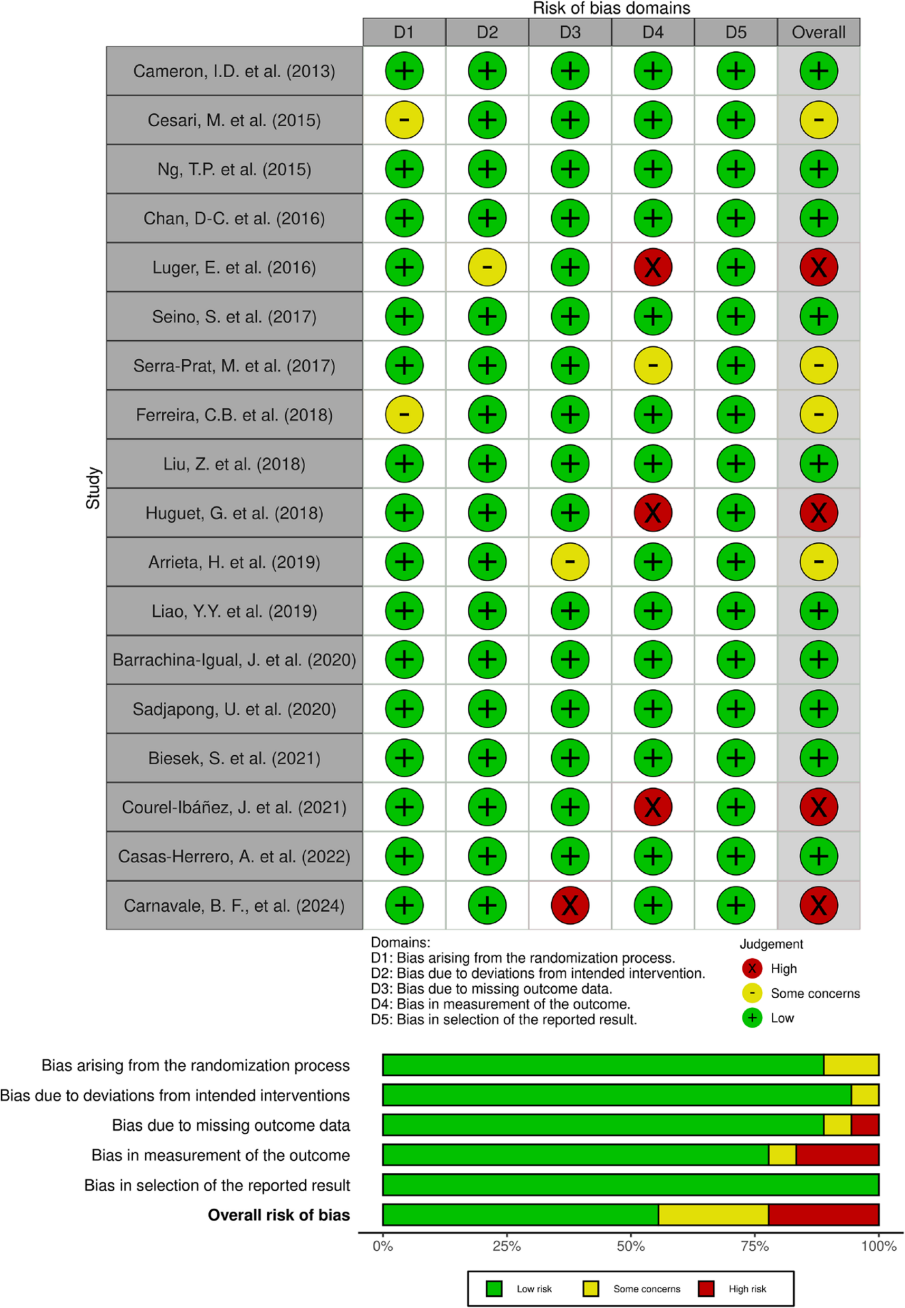
Risk of bias (RoB2.0 assessments) traffic light plot and summary plot of the multicomponent exercise RCTs is placed here

**Fig. 3:**
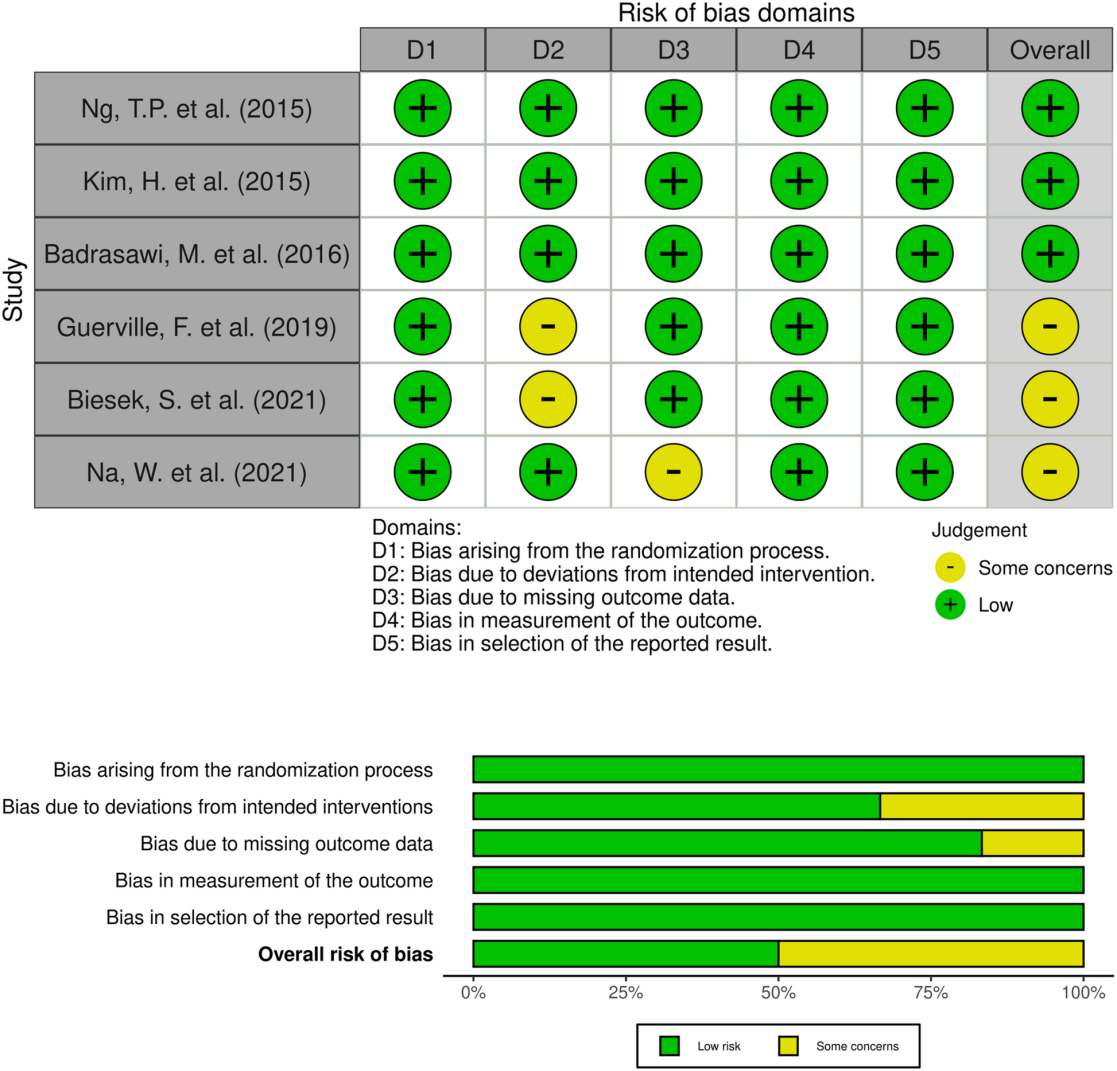
Risk of bias (RoB2.0 assessments) traffic light plot and summary plot of the nutritional intervention RCTs is placed here

Each of the included studies was evaluated for risk of bias using RoB 2.0 tool for RCTs and ROBIN-I tool for CCTs. Figures 2 and 3 provide a summary of these evaluations for multicomponent exercise RCTs and nutritional intervention RCTs, respectively.

#### Risk of bias in the multicomponent exercise studies

In terms of the overall risk of bias, there were concerns about bias in half of the multicomponent exercise RCTs (8/18); four of these were rated as having a high risk of bias, and four studies had some concern risk of bias. We classified three studies with a high overall risk of bias in the outcome measurement and one study with missing outcome data. Luger, E. et. al., 2016 [39] study was constrained by the fact that the outcome was assessed during home visits by non-professional volunteers who were aware of the intervention received by study participants. For Huguet, G. et al., 2018 [40] and Courel-Ibáñez, J. et al., 2021 [41], there was a potential risk of bias in the outcome measurement because the investigator teams who were both outcome assessors and intervention providers, aware of the group patients. Carnavale, B. F., et al. (2024) [42] had a high risk of bias due to the high drop-out percentage in the control arm (45%) compared to the intervention arm (20%) with a limited sample size (20 per arm). Two studies were classified as having some concern about the risk of bias due to baseline differences (Cesari, M. et al., 2014) [43] and no information about allocation sequences that were either concealed or randomized (Ferreira, C.B. et al., 2018) [44]. Arrieta, H. et al., 2019 [45] reported dropout rates of 24.6% and 29.1% for the intervention and control groups, respectively, which raised some concern about the risk of bias due to missing outcome data. Serra-prat, M. et al., 2017 [46] had some concerns about the risk of bias because outcomes assessors was not blinded to the intervention group. Two multicomponent exercise CCTs by Seesen, M. et al., 2020 [47] and Chiu, T. Y. et al., 2022 [48] were rated as having a moderate overall risk of bias due to moderate concerns of bias in two domains; (1) bias due to residual confounders from high baseline physical performances in the exercise group (Seesen, M. et al., 2020 [47]) and in the control group (Chiu, T. Y. et al., 2022 [48]); and (2) bias in measurements of outcome because outcome assessors were probably aware of the intervention received by study participants. Another CCT by García-Vigara, A. et al. (2024) [49] was rated as having a low overall risk of bias, with moderate concerns similarly related to outcome measurement bias due to assessor awareness of participant intervention.

#### Risk of bias in the nutritional intervention studies

In term of overall risk of bias, three of six nutritional intervention RCTs were assessed as having some concern overall risk of bias. Biesek, S. et al., 2021 [50] was rated as having some concerns about the risk of bias due to deviations from the intended interventions (the effect of adhering to an intervention) because of a lower average adherence rate to the use of protein and/or isoenergetic supplementation, which ranged from 50.1% to 74.9% of that prescribed, as well as Guerville, F. et al., 2019 [51] due to no information about the adherence to the intervention. Na, W. et al., 2021 [52] was rated as having some concerns about the risk of bias due to missing outcome data in placebo group (dropout 19.4%). One CCT, Seesen, M. et al., 2020 [47], on nutritional intervention had moderate risk of bias due to residual confounders and bias measurement of outcomes as described above.

### Results of syntheses

#### The multicomponent exercise studies

Twenty-one included studies (18 RCTs and three CCT) between 2013 and 2024 investigated the effect of multicomponent exercises on improving physical frailty in community-dwelling older adults. Studies were conducted in diverse geolocation settings; seven in Spain [40, 41, 45, 46, 49, 53, 54], three in Taiwan [48, 55, 56], two in the United States [43, 57], three in Brazil [42, 44, 50], two in Thailand [12, 47], and one each in Australia [58], Austria [58], Japan [59], and Singapore [60]. Nine studies [41, 44, 45, 48, 50, 53, 55, 56, 59] offered center-based exercise training, four studies [39, 46, 54, 58] offered home-based exercise training, and six studies [12, 40, 43, 47, 57, 60] included both center-based and home-based training programs. In term of the length of exercise intervention, nine RCTs [12, 39, 42, 44, 50, 53, 54, 56, 59] and two CCTs [47, 48] offered 3 months programs; Five RCTs [40, 41, 45, 55, 60] offered more than 3 to 6 months programs; and Four RCTs [43, 46, 57, 58] and one CCT [49] offered ≥ 12 months programs. All of the purposed exercise programs combined strength training with balance, flexibility, or endurance training. The most common programs were the combination of all training (seven RCTs [41–44, 53, 54, 56] and two CCTs [48, 49]) and strength, balance, and endurance training (six RCTs [12, 40, 46, 50, 58, 60] and one CCT [47]); followed by strength, balance, and flexibility training (two RCTs [55, 57]); and dual programs that combined strength training with either balance (one RCT [45]) or endurance training (two RCTs [39, 59]).

In terms of participant characteristics, all studies reported on study participant age and generally included participants in their 70 s and 80 s. All included studies reported gender proportion except Ferreira, C.B. et al. (2018) [44]. The majority of participants in the included studies were female, ranging from 53 to 84%, except for the study by Biesek, S. et al., 2021 [50] and García-Vigara, A. et al. (2024) [49], which enrolled only female older adults. Only one RCT, Seino, S. et al., 2017 [59], reported that the majority of participants were male (69%). Most of the included studies assessed frailty status using CHS frailty index, except the study by Luger, E. et al., 2016 [39] that used SHARE-FI index.

**Fig. 4:**
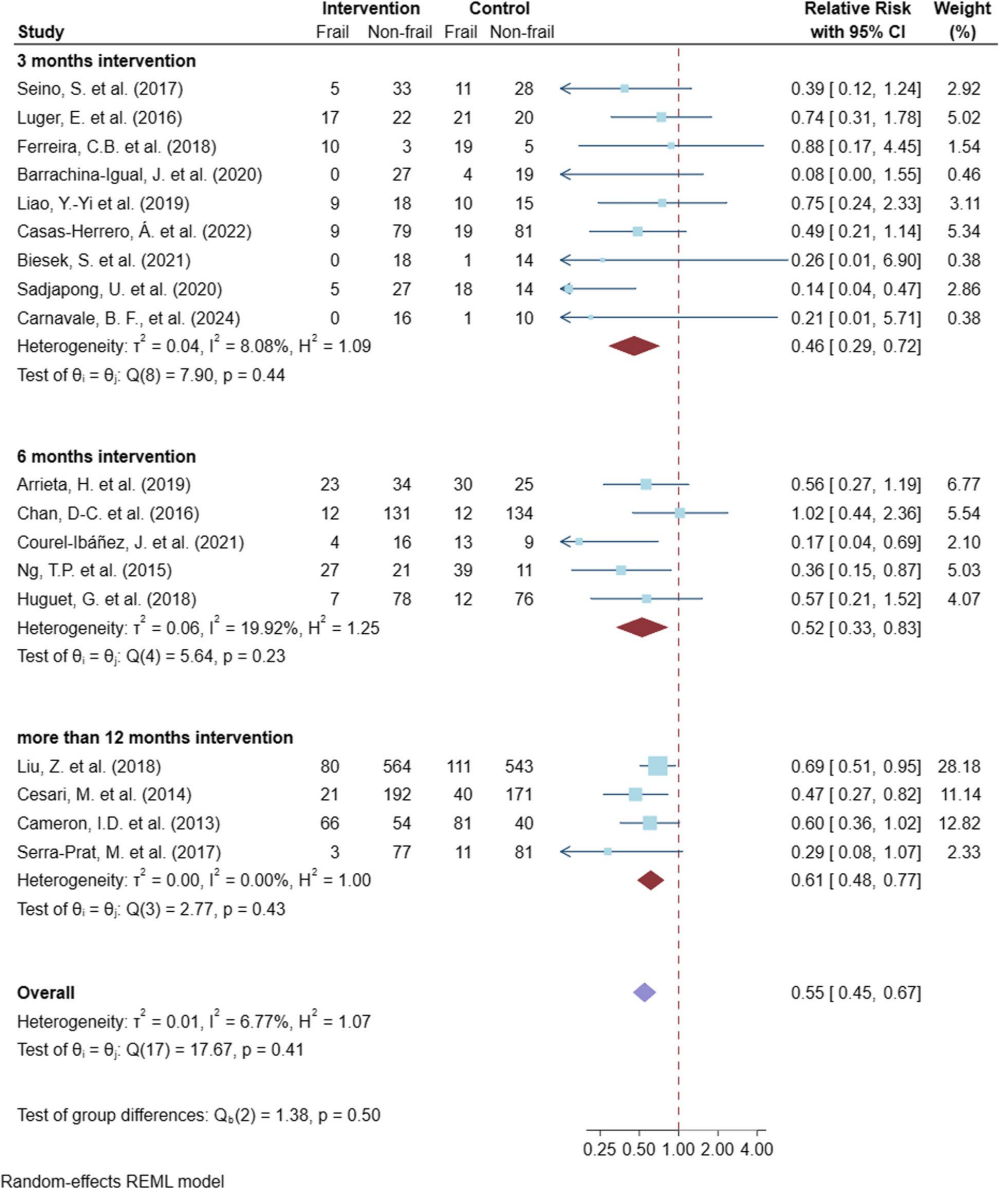
Meta-analysis of the multicomponent exercise interventions for improving physical frailty and subgroup analysis by lengths of intervention is placed here

For the meta-analysis of the multicomponent exercises, the CCT studies by Seesen, M. et al., 2020 [47], Chiu, T. Y. et al., 2022 [48], and García-Vigara, A. et al. (2024) [49] were excluded from the primary analysis because of concerns regarding the heterogeneity of the study design. Pooled analysis of eighteenth multicomponent exercise RCTs, including a total of 3457 older adults, the multicomponent exercises showed a clinically significant reduction in frailty risk by relative change 55% times (95% CI 45% to 67%, *p* value < 0.001; moderate certainty of evidence) as shown in Fig. 4. The subgroup meta-analysis by the length of intervention also showed the robustness of the efficacy of the multicomponent exercises on a decrease of frailty risk. The statistical heterogeneity was not important for the overall analysis (*I*^*2*^ 6.77%) and the subgroups of the length of intervention (Fig. 4). In addition, the robustness of this result was supported by the outcomes of other sensitivity analyses, which included both RCTs and CCTs, the subgroup analysis by study bases and types of exercises. The details of all sensitivity analyses are presented in Supplementary Table S3.

#### The nutritional intervention studies

Between 2015 and 2021, Six RCTs [50–52, 60–62] and one CCT [47] investigated the effect of nutritional interventions on decreasing physical frailty risk in community-dwelling older adults. Studies were conducted in diverse geolocation settings, one each in Brazil [50], France [51], Japan [62], Korea [52], Malaysia [61], Singapore [60], and Thailand [47]. Interventions were based on macronutrients in two RCTs (protein and fat) [50, 62], micronutrients in one CCT (Anthocyanin) [47] and two RCTs (L-carnitine and Omega-3 fatty acids) [51, 61], and both macronutrients (Carbohydrate, protein, and fat) and micronutrients (multivitamins and minerals) in two RCT [52, 60]. The largest study was RCT in 689 France community-dwelling older adults that provided macronutrient (omega-3 supplementation) for 3 years [51]. One CCT [47] on anthocyanin and one RCT [60] on both macronutrients and micronutrients offered 6 months of supplementation. Two RCTs [50, 62] on macronutrients, one RCT on micronutrients [61], one RCT [52] on both macronutrients and micronutrients offered less than 3 months of supplementation. The analyses of nutritional interventions are categorized into two main intervention groups, including the studies on macronutrients with and without micronutrient supplements and the studies on micronutrients only. The purpose of a macronutrient supplement is the use of food fortification (energy-dense) and protein-energy supplementation to improve frailty. On the other hand, a micronutrient supplement is taken as a supplement to suppress inflammatory processes and maintain the normal functioning of the immune system rather than provide additional energy.

In term of participant characteristics, all studies reported on study participant age and generally included participants in their 70 s and 80 s. Two RCTs [50, 62] recruited only female older adults. The other studies had female participants as the majority, ranging from 63.3% to 72.3%, except for the study by Badrasawi, M. et al., 2016 [61], in which female participants were 46.2%. Regarding the baseline nutritional state, none of the included studies have specific eligibility criteria for screening nutritional status or malnourished individuals. Of two RCTs examined combination of macronutrients and micronutrients, the RCT conducted by Na, W. et al., 2021 [52] provided information on the participants’ mean (± SD) Mini Nutritional Assessment (MNA) score of 19.8 (± 3.1), indicating the range considered at risk of malnutrition. Another RCT [60] was considered a non-malnourished group due to the low reported prevalence of unintentional weight loss (4% in the intervention group and 6% in the control group) and the participants’ anthropometric measurements averaging within the normal range. Three studies [47, 51, 61] on micronutrient interventions were also considered a non-malnourished population because most participants were non-frail, and the reported average anthropometric measurements were within the normal range. In contrast, two RCTs [50, 62] in the subgroup of macronutrient interventions recruited only pre-frail and frail older women. The participants in these studies were considered to have a higher degree of frailty severity and a significant risk of malnutrition due to the high prevalence of unintentional weight loss (ranging from 16.7% to 62.5%). In terms of physical activity, participants in two RCTs that provided a combination of micronutrients and macronutrients reported high levels of physical activity. The RCT by Na, W., et al., 2021 [52] reported that 85.5% of the participants engaged in regular exercise more than four times per week. Another study by Ng, T.P. et al., 2015 [60] reported that the nutritional intervention arm had a mean (± SD) daily physical activity of 279 (± 139) minutes, which was significantly higher than the other arms. The participants' characteristics from three studies on micronutrients and one RCT on macronutrients were also considered normal physical activity due to the small prevalence of low physical activity (ranging from 5.6% to 11%) [50] and their reported average assessments of physical performance within the normal range [47, 51, 61]. Only one RCT by Kim, H. et al., 2015 [62] on macronutrients studied the population with a high prevalence of low physical activity (ranging from 90.9% to 93.8%). All included studies assessed frailty status using the CHS frailty index.

**Fig. 5:**
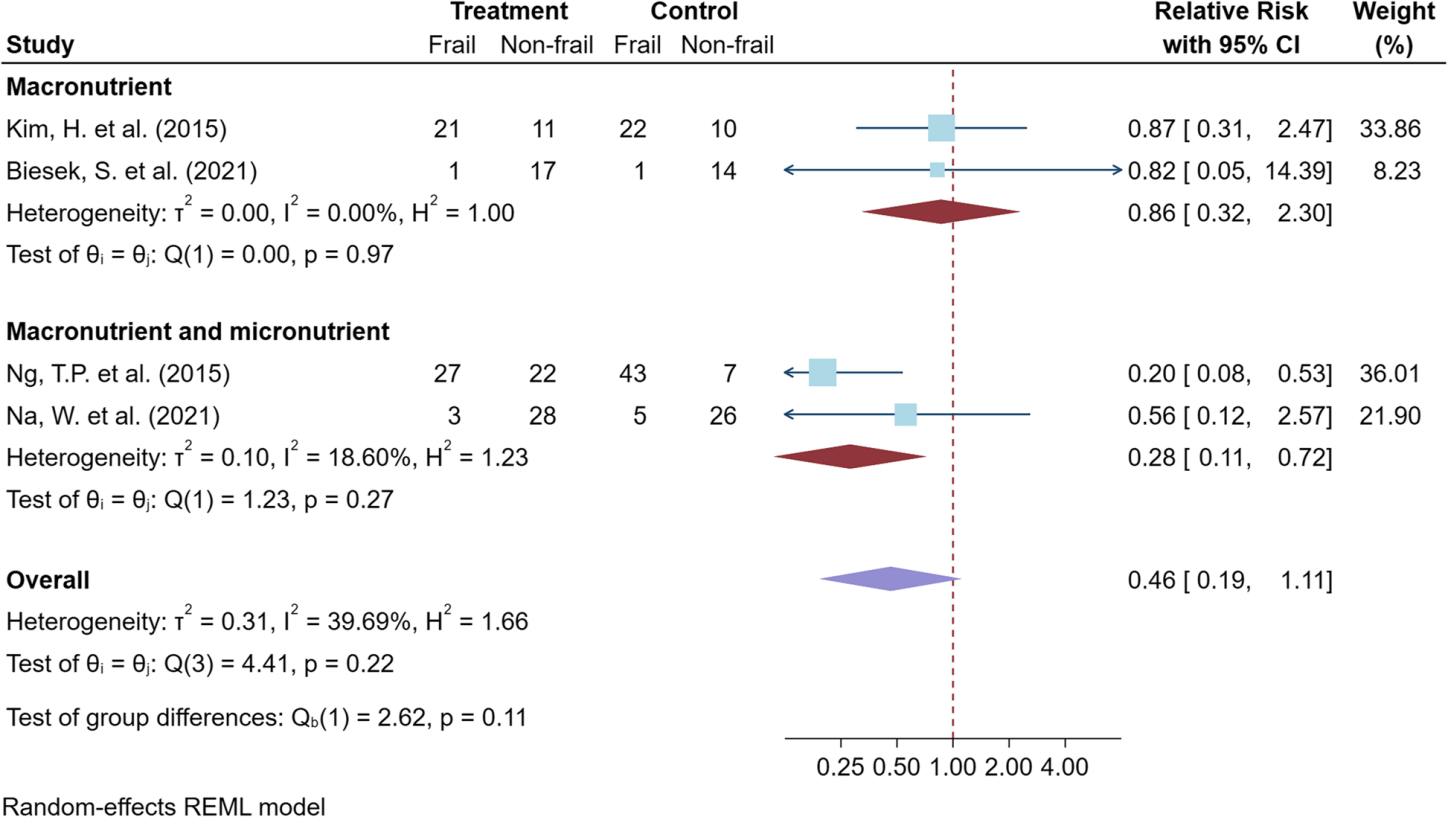
Meta-analysis of the macronutrient supplements for improving physical frailty and subgroup analysis is placed here

**Fig. 6:**
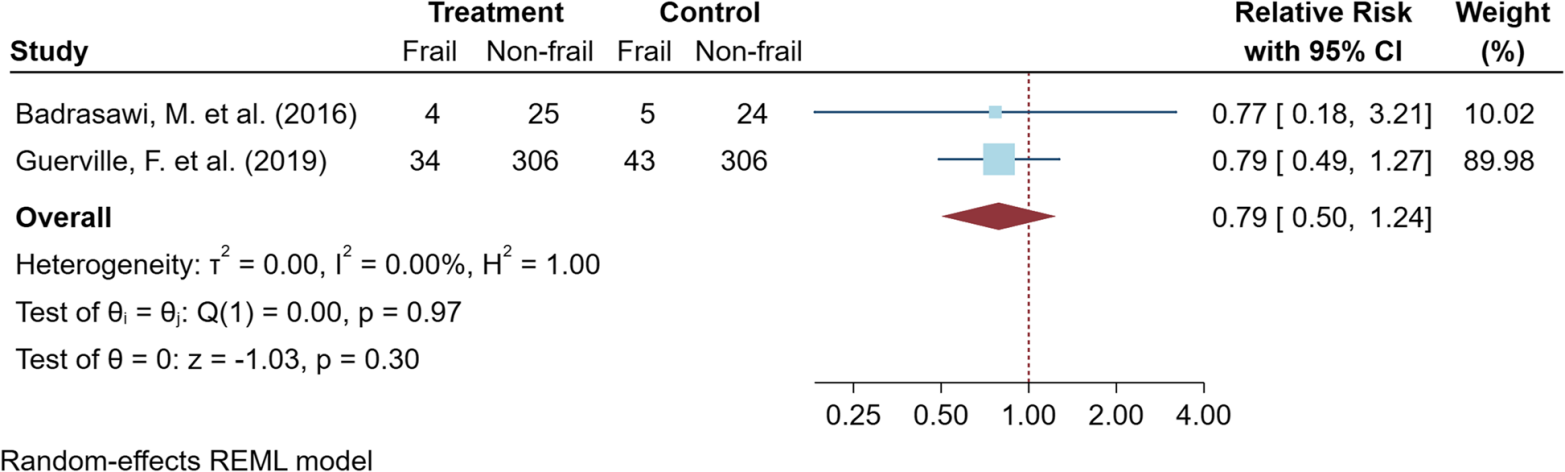
Meta-analysis of the micronutrient supplements for improving physical frailty is placed here

For the meta-analysis of the nutritional interventions, the CCT study by Seesen, M. et al., 2020 [47] was excluded from the primary analysis because of concerns regarding the heterogeneity of the study design. Pooled analysis of four RCTs provided macronutrient with and without micronutrient supplements, including a total of 258 older adults, did not show a statistically significant decrease in frailty risk by relative change 49% times (95% CI 19% to 111%, *p* value = 0.065; very low certainty of evidence) as shown in Fig. 5. The heterogeneity of the meta-analysis was moderate, but not statistically significant (*I*^*2*^ 39.7%, *p* value = 0.22). The different lengths and types of nutritional intervention could contribute to this heterogeneity, as shown by the sensitivity analysis (Table S4). Only subgroup analysis of two RCTs, offered the combination of macronutrients and micronutrients, showed a clinically significant decrease in frailty risk by relative change 28% times (95% CI 11% to 72%, *p* value = 0.008; low certainty of evidence) with low and non-significant heterogeneity (*I*^*2*^ 18.6%, *p* value = 0.268). For the studies on micronutrient supplements, the meta-analysis of two RCTs did not show a statistically significant decrease in frailty risk as presented in Fig. 6.

### Reporting biases

#### The multicomponent exercise studies

**Fig. 7:**
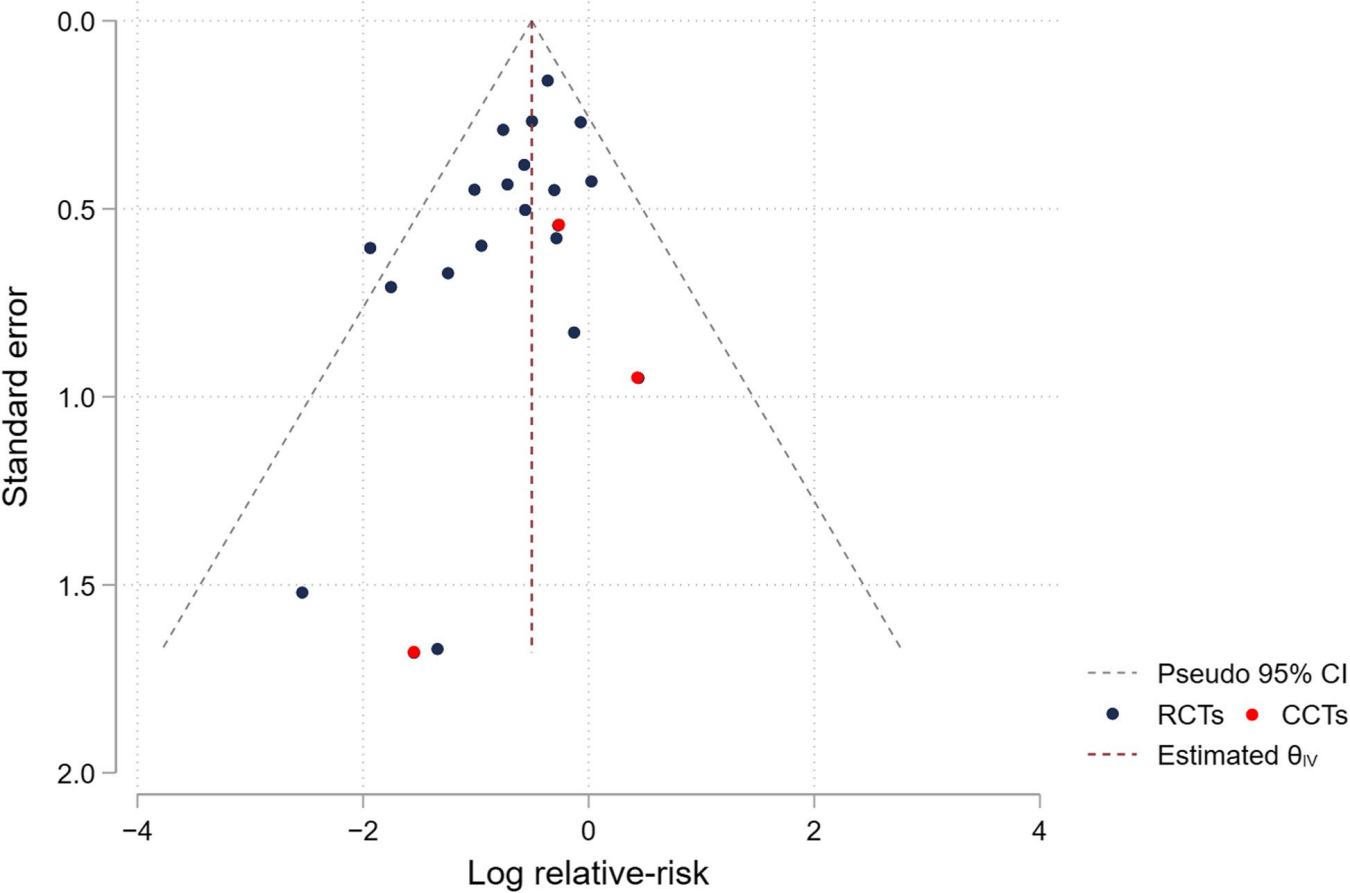
Funnel plots of the eligible multicomponent exercise studies is placed here

To assess reporting bias, we generated funnel plots for the meta-analysis of the multicomponent exercise studies, including 21 eligible studies (Fig. 7). The asymmetry pattern of the funnel plot was detected due to two small studies, which favored the multicomponent exercise interventions. We performed the additional statistical analyses for investigating funnel plot asymmetry and small-study effects. The results of the regression-based tests, Egger's and Harbord test, did not demonstrate a statistically significant effect from a small study (*p*-values = 0.076 and 0.075, respectively). The sensitivity analysis of missing studies was also performed using trim and fill method (Figure S1). The adjusted effect size of the multicomponent exercise interventions (adjusted RR 0.64, 95%CI 0.54–0.72; *p*-value < 0.001) was slightly reduced compared to the pooled estimate from the meta-analysis of all eligible studies (pooled RR 0.59, 95%CI 0.50–0.71; *p*-value < 0.001). With these collective findings, we therefore conclude that the meta-analysis results shown in Fig. 4 are likely robust to any small study/publication bias.

#### The nutritional intervention studies

**Fig. 8:**
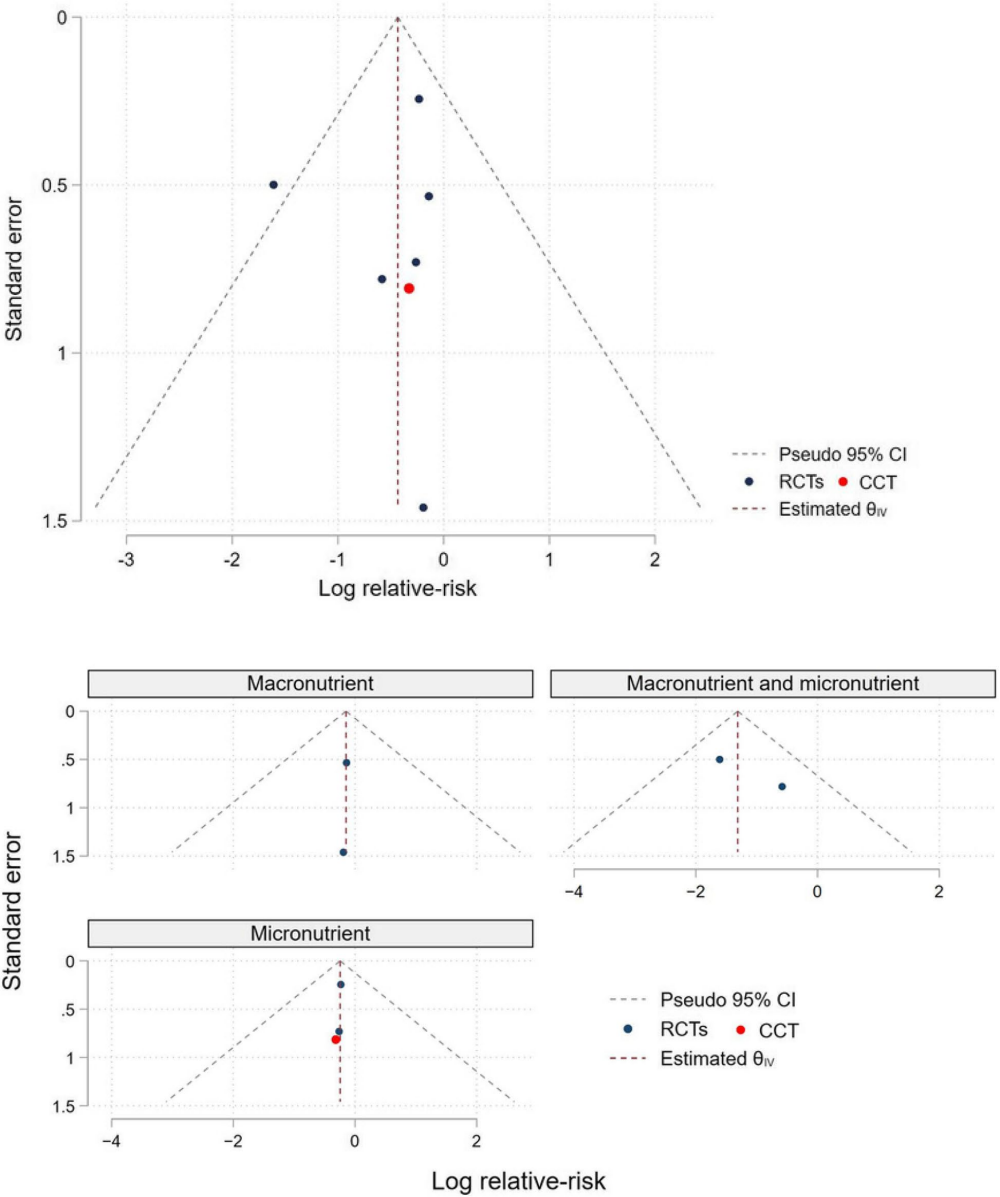
Funnel plots of the eligible nutritional intervention studies and subgroup plots by types of nutritional intervention is placed here

To assess reporting bias, we generated funnel plots for the meta-analysis of the nutritional intervention studies, including 7 eligible studies (Fig. 8). Due to a small number of the eligible studies (< 10 studies), we did not perform the additional statistical analyses for assessing small-study effects. Inspection of the funnel plots of all eligible studies and the subgroups of intervention types showed a symmetry pattern. We therefore conclude that there is no evidence of reporting bias from the meta-analysis of the nutritional intervention studies.

### Certainty of evidence

Table 1 presents the summary of findings derived from the studies on multicomponent exercise and nutritional intervention, together with an assessment of the certainty of the evidence.

**Table 1:** Summary of findings

**Population:** Community-dwelling older adults **Intervention:** Multicomponent exercises or nutritional interventions **Comparison:** Usual care or placebo (nutritional interventions) **Outcome:** Incidence of frailty				
**Interventions**	**Number of participants (studies)**	**Relative effect (95%CI)**	**Certainty of the evidence (GRADE)**	**Comments**
**Multicomponent exercises**				
	3457 (19 studies)	**RR 0.59 **(0.50 to 0.71)	⊕ ⊕ ⊕ ◯ **Moderate**^a^Due to risk of bias	Probably decreases the incidence of frailty
*Durations of intervention*				
3 months	608 (9 RCTs)	**RR 0.46**(0.29 to 0.72)	⊕ ⊕ ⊕ ◯ **Moderate**^b^Due to risk of bias	Probably decreases the incidence of frailty
6 months	714 (5 RCTs)	**RR 0.52 **(0.33 to 0.83)	⊕ ⊕ ⊕ ◯ **Moderate**^c^Due to risk of bias	Probably decreases the incidence of frailty
≥ 12 months	2135 (4 RCTs)	**RR 0.61**(0.48 to 0.77)	⊕ ⊕ ⊕ ◯ **Moderate**^d^Due to risk of bias	Probably decreases the incidence of frailty
**Nutritional interventions**				
	1005 (6 RCTs)	**RR 0.59 **(0.34 to 1.03)	⊕ ◯◯◯ **Very low**^e,f,g^Due to risk of bias, inconsistency, and indirectness	May decreases the incidence of frailty
*Types of nutrients*				
Macronutrients and Micronutrients	161 (2 RCTs)	**RR 0.28 **(0.11 to 0.72)	⊕ ⊕ ⊕ ◯ **Moderate**^h^Due to inconsistency	Probably decreases the incidence of frailty in individuals with high level of physical activity
Macronutrients	97 (2 RCTs)	**RR 0.86 **(0.32 to 2.30)	⊕ ⊕ ◯◯ **Low**^i^Due to serious imprecision	There may be little or no difference in frailty incidence
Micronutrients	747 (2 RCT and 1 CCT)	**RR 0.79 **(0.50 to 1.24)	⊕ ⊕ ⊕ ◯ **Moderate**^j^Due to indirectness	There may be little or no difference in frailty incidence

*Abbreviations*: *CI* Confidence interval, *RR* Risk ratio, *GRADE* GRADE Working Group grades of evidence

Explanations

^a^High risk of bias due to having three RCTs with high risk of bias in the outcome measurement, one RCT with high risk of bias in the missing outcome data, and four RCTs with some concern risk of bias (baseline differences in one RCT, no information about allocation sequences in one RCT, a high drop-out percentage in one RCT, and the outcome assessors being aware of the intervention group in one RCT)

^b^High risk of bias due to having one RCT with high risk of bias in the outcome measurement, one RCT with high risk of bias in the missing outcome data, and one RCT with some concern risk of bias due to no information about allocation sequences

^c^High risk of bias due to having two RCTs with high risk of bias in the outcome measurement and one RCT with some concern risk of bias due to a high drop-out percentage

^d^Moderate risk of bias due to having one RCT with some concern about the risk of bias due to baseline differences and one RCT with some concern risk of bias due to the outcome assessors being aware of the intervention group

^e^Moderate risk of bias due to having three RCTs with some concern risk of bias due to low adherence rate in the intervention group, not report on adherence rates, and high drop-out percentage in the placebo group

^f^Moderate inconsistency due to intervention variability (moderate heterogeneity I^2^ = 36.3%, *p-*value = 0.248, point estimates and confidence intervals vary considerably)

^g^Indirectness of evidence due to intervention variability (types of nutrients)

^h^Some inconsistency due to duration of intervention and baseline frailty status (low heterogeneity I^2^ = 18.6%, *p-*value = 0.268)

^i^Serious imprecision due to few eligible studies and participants

^j^Indirectness of evidence due to intervention variability (types of micronutrients)

## Discussion

Our findings demonstrated the benefits of multicomponent exercises for reducing frailty in community-dwelling older adults. In addition, the benefits of multicomponent exercises were robustly demonstrated across all subgroup analyses by study design, study population, intervention durations, and exercise types. Furthermore, the subgroup meta-analysis of two RCTs on combinations of macronutrients and micronutrients showed a clinically significant decrease in the occurrence of frailty with moderate certainty of evidence due to some concerns about the consistency due to the different durations of the interventions and the baseline frailty status. However, the pooled estimate of the other subgroup analyses, including the studies on either macronutrients or micronutrients, did not show statistically significant benefits for frailty reduction.

From our findings, the efficacy of the multicomponent exercise interventions is substantiated by previously published analyses. A previous network meta-analysis revealed that physical activity, multicomponent interventions, and nutritional interventions were associated with a reduction in frailty among participants aged 60 years and older, as evidenced by pooled standard mean differences of frailty scores. Resistance (strength) training was identified as the most effective intervention, following by aerobic (endurance) training [17]. Another network meta-analysis also demonstrated the efficacy of physical activity (any form of exercise) as an intervention for reducing standard mean differences of frailty scores comparing to a routine care. [16] In addition, our findings from the subgroup analyses of exercise types indicated that multicomponent programs consisting of strength and endurance training had the potential to reduce frailty risk significantly (Table S3). Considering that low physical activity and muscle weakness are the primary indicators of physical frailty, it is reasonable that strength and endurance exercise be prioritized to mitigate the risk of frailty in the previous studies. According to the established evidence and our results, we recommend that multicomponent exercise regimens, which include both strength and endurance training, be considered the primary approach for frailty management. In a previous diagnostic study on sarcopenia, anterior thigh muscle thickness showed the most significant decline with aging and was the measurement most strongly related to body mass and height. Additionally, the sonographic thigh adjustment ratio values were negatively correlated with the Chair Stand Test and the Timed Up and Go Test [63], both of which assess functions that can be improved by exercise components targeting strength [64] and balance [65], respectively.

The efficacy of nutritional intervention alone in improving frailty is controversial, especially in cases where frailty is observed without unintentional weight loss, malnutrition, or sarcopenia. Several guidelines on frailty management recommended macronutrient supplementation for only pre-frail or frail patients when they were experiencing unintentional weight loss or undernutrition, hospitalized, or undergoing exercise programs [3, 66, 67]. The significant efficacy of macronutrients and micronutrients in our analysis could be attributed to the fact that their participants' characteristics were mainly robust and pre-frail older adults with high levels of physical activity. The absence of benefits from macronutrient supplements in the included studies and our meta-analysis could be attributed to the high level of frailty severity observed in the study population, though they were considered to have a significant risk of malnutrition. In the same way, micronutrient supplementation, particularly essential vitamins and trace elements, for improving frailty or physical performance is not recommended unless a deficiency is present [3, 66, 67]. The roles of micronutrient supplements for frailty management are to reduce systemic inflammatory processes via regulation of oxidative stress and to maintain the normal functioning of the immune system. Due to the multifaceted characters of frailty syndrome, depending solely on nutritional intervention is insufficient for mitigating frailty, particularly in pre-frail older adults who are not malnourished, and frail older adults. To improve functional ability, other multicomponent interventions, such as a multicomponent exercise program, psychological intervention, and health education, should be incorporated with appropriate nutritional supplements. Previous research findings also provided support for the use of multicomponent methods, particularly the combination of exercise and appropriate nutritional supplements. One systematic review and meta-analysis, included the conflicting studies’ results between nonsignificant and significant effects, illustrated that protein supplement combined with muscle strengthening exercise is beneficial for sarcopenia and frailty in terms of increasing mass and strengthening gain [68]. Furthermore, the systematic review examining the efficacy of nutrition intervention supported that multicomponent interventions were considered more effective than nutrition intervention alone for improving frailty [69].

The review demonstrated several strengths in its methodology and results, which adhered to the recommended guidelines outlined by Cochrane [32] and PRISMA [25] for conducting and reporting a systematic review and meta-analysis. This involved implementing systematic search strategies, thoroughly screening relevant literature, extracting data, and evaluating the risk of bias. The review processes and assessment of bias were conducted by clinicians (W.S., N.B., and K.P.) and healthcare professionals (P.S.) who have experience in conducting meta-analysis studies and regularly work with older adults in both community and clinical contexts. However, it is important to exercise caution when interpreting this study due to the limitations described. First, the number of studies included in this review was relatively small compared to the existing literature [16, 17]. Although our review applied broad search terms without particular study design limits, it is conceivable that we may have inadvertently missed eligible studies that were exclusively available in alternative databases or remained unpublished [70, 71]. Second, as previously mentioned, our study primarily examined the relative risk of frailty rather than changes in frailty scores. There were studies that met the inclusion criteria in the analysis but did not provide information on the incidence of frailty, and we are unable to contact the authors of these studies to obtain the relevant data. Third, although there were many studies on the combination of both exercise and nutritional supplements, a systematic review, and a meta-analysis of them were not performed in our study due to the substantial and pronounced heterogeneity across multiple aspects of the studies, including methodological approaches, types of interventions employed, and durations of the interventions. For the analysis of the multicomponent exercise studies, some studies included in this study exhibited a moderate to high overall risk of bias. Four of the 17 multicomponent exercise RCTs that were included had some concerns or a high risk of bias in the measurement of the outcome because the outcome assessors probably knew which arm the participants were in. This could have affected how the outcome data was measured. However, it could be argued that the awareness of the study groups may not cause a serious bias because the diagnosis criteria for frailty are based on four objective measurements and only one subjective question on self-reported exhaustion. For the analysis of the studies on nutritional interventions, half of them had only some concerns about the risk of bias, either in baseline imbalance, deviation from the intended intervention, or missing outcome data. Nevertheless, with the small number of eligible studies on nutritional supplements and their heterogeneity of nutrients, the results from the meta-analysis of these studies should be interpreted with acknowledgement of these limitations. Although the intervention patterns could be discriminated between studies providing macronutrients, micronutrients, and both, the nutrient characteristics and the quantity of nutrients varied widely across studies, particularly in the studies offering micronutrients. Hence, the pool estimate of these studies may not represent a true marginal effect of nutritional interventions on reducing risk of frailty.

Sarcopenia, the age-related loss of muscle mass and function, is a physical component of frailty. It is influenced by genetic and lifestyle factors throughout the course of life, as well as by current risk factors. The treatment and prevention of sarcopenia are expected to become standard components of clinical practice [72]. The results of our study indicate that multicomponent exercise is a promising intervention for mitigating the risk of frailty, followed by nutritional supplements with a combination of macronutrients and micronutrients. The results obtained from the included studies on multicomponent exercises indicate that both short-term and long-term programs and different types of exercise demonstrate similar efficacy in mitigating frailty. Given the dynamic nature of frailty, it is plausible for older adults who are robust to experience a regression back into a state of frailty. The potential advantages of prolonged intervention must be balanced against considerations such as patient adherence and the cost-effectiveness of the intervention. Therefore, the primary emphasis of the practice should be placed on strategies to enhance self-efficacy and ensure its long-term viability. To achieve it, exercise programs should be tailored and redesigned for each specific cultural and social context. Healthcare practitioners could use our summaries as evidence-based components, doses, and duration of interventions to alleviate frailty [73]. Since the subgroup analysis showed no difference in terms of efficacy between center-based and home-based interventions, a combination of home-based and center-based programs would be more suggestive. Engaging in a center-based program makes exercise a social activity, which promotes social engagement and independence. Also, engaging in home-based exercise makes it easier to fit an exercise program into their routine schedule, which increases self-efficacy and long-term viability. Regarding the implementation, we suggest that non-pharmacological treatments for frailty should incorporate multicomponent approaches that incorporate strength and endurance (aerobic) exercise and appropriate nutritional supplements in order to optimize therapeutic efficacy and mitigate the occurrence of frailty. The investigation of personalized multicomponent therapies holds promise for tackling frailty problems in community-dwelling older patients, both in terms of treatment and prevention. Despite the constraints posed by the limited number of studies and the heterogeneity of interventions and treatment protocols, the analysis of multicomponent therapies was not investigated in this study. The clinical significance of multicomponent approaches for frailty remains uncertain and necessitates further exploration in future research.

## Conclusions

Multicomponent exercises can effectively improve physical frailty, regardless of the duration and types of the activities. Nevertheless, the efficacy of nutritional supplements remains inconclusive. Personalized multicomponent approaches that incorporate both exercises and nutritional supplements have promise for enhancing effectiveness in reducing frailty, thus warranting further investigation.

## Supplementary Information

Supplementary Material 1.

Supplementary Material 2.

Supplementary Material 3.

## Data Availability

No datasets were generated or analysed during the current study.

## Abbreviations

CCTs: Controlled clinical trials
CHS: Cardiovascular Health Study
CI: Confidence interval
CINAHL: Cumulative Index to Nursing and Allied Health
GRADEL: GRADE Working Group grades of evidence
MNA: Mini Nutritional Assessment
PRISMA: Preferred Reporting Items for Systematic Reviews and Meta-Analysis
RCTs: Randomized controlled trials
REML: Restricted Maximum Likelihood
RR: Risk ratio
SD: Standard deviation
