# Supplementary material for: Impact of multicomponent exercise and nutritional supplement interventions for improving physical frailty in community-dwelling older adults: a systematic review and meta-analysis
Source: BMC Geriatr. 2024 Nov 18;24:958. doi: 10.1186/s12877-024-05551-8 (PMC11571505; doi:10.1186/s12877-024-05551-8)
Supplement: Supplementary file 2 — Supplementary Material 2. [file 12877_2024_5551_MOESM2_ESM.docx]

**Supplementary Materials**

**Page**

**Search strategy** 1

**Table S1.** The characteristics of the eligible multicomponent exercise studies. 2 - 6

**Table S2.** The characteristics of the eligible nutritional intervention studies. 7 - 8

**Table S3.** Subgroup and sensitivity analyses of multicomponent exercise studies 9

**Table S4.** Subgroup and sensitivity analyses of nutritional studies 10

**Figure S1.** Funnel plot of the multicomponent excercises studies with trim and fill method 11

This supplementary material has been provided by the authors to give readers additional information about their work.

**Search strategy**

| **PubMed** | |
| --- | --- |
| **Search term** | (Older adults OR (Aged[MeSH]) OR Elderly) AND ((Exercise[MeSH]) OR Physical activity* OR Nutritional* OR (Exercise therapy[MeSH])) AND ((Frailty[MeSH]) OR Debility OR Frail*) |
| **Filter** | **Text availability:** Full text  **Article type:** Clinical trial, Comparative study, Randomized Controlled Trial  **Article Language:** English  **Year:** 2000 to present |
| **Initial search** | (n = 780; Sep 19, 2022) |
| **Last search** | (n = 1077; Oct 10, 2024) |
| **EMBASE** | |
| **Search term** | ('older adults'/exp OR 'older adults' OR (older AND ('adults'/exp OR adults)) OR 'elderly'/exp OR elderly OR 'aged'/exp OR aged) AND (exercise* OR nutritional* OR physical) AND activity* AND (frailty* OR 'debility'/exp OR debility OR frail*) AND [2000-2023]/py AND ('clinical trial'/de OR 'comparative study'/de OR 'controlled clinical trial'/de OR 'randomized controlled trial'/de) AND [english]/lim |
| **Filter** | **Publication type:** Article  **Study type:** Clinical trial, Comparative study, Controlled Clinical trial, Randomized Controlled Trial  **Article Language:** English  **Year:** 2000 to present |
| **Initial search** | (n = 745; Sep 19, 2022) |
| **Last search** | (n = 1060; Oct 10, 2024) |
| **Cumulative Index to Nursing and Allied Health (CINAHL)** | |
| **Search term** | (Older adults OR Elderly OR Aged) AND (Exercise* OR Nutritional* OR Physical activity*) AND (Frailty* OR Debility OR Frail*) |
| **Filter** | **Publication type:** Academic Journal  **Article Language:** English  **Year:** 2000 to present |
| **Initial search** | (n = 2579; Sep 19, 2022) |
| **Last search** | (n = 3,190; Oct 10, 2024) |

**Table S1.** The characteristics of the eligible multicomponent exercise studies.

| **Author (year), Country** | **Mean age,**  **year (±SD)** | **Participants**  **(Intervention/**  **Control)** | **Study designs**  **(Study base)** | **Intervention** | **Control** | **Outcome measures** |
| --- | --- | --- | --- | --- | --- | --- |
| Luger, E. et al. (2016), Austria | 82.6 (±8.0) | 80 older adults  ≥ 65 years  (39/41) | Single-center, RCT, assessors-blinded  (Home-based) | **Mode:** Strength & Endurance training  **Duration**: 30 min-session  **Frequency**: 7/week  **Length**: 3 months  **Dropout**: 13% | Social Support Group  **Dropout**: 22% | **Tool**: SHARE-FI index  **Follow-up time**: 3 months |
| Seino, S. et al. (2017), Japan | 74.6 (±5.4) | 77 pre-frail and frail older adults  65-84 years  (38/39) | Single-center, cross-over RCT, assessors-blinded  (Center-based) | **Mode:** Strength & Resistance training  **Duration**: 60 min-session  **Frequency**: 2/week  **Length**: 3 months  **Dropout**: 11% | Usual care  **Dropout**: 9% | **Tool**: CHS Frailty index  **Follow-up time**: 3 months |
| Ferreira, C.B. et al. (2018), Brazil | 76.1 (±7.7) | 45 older adults  ≥ 60 years  (15/30) | Single-center, RCT, assessors-blinded  (Center-based) | **Mode:** Strength & Resistance & Flexibility training  **Duration**: 40 min-session  **Frequency**: 3/week  **Length**: 3 months  **Dropout**: 13% | Usual care  **Dropout**: 20% | **Tool**: CHS Frailty index  **Follow-up time**: 3 months |
| Liao, Y.-Yi et al., Taiwan | 81.8 (±7.0) | 61 pre-frail older adults  65-90 years  (31/30) | Single-center, RCT, assessors-blinded  (Center-based) | **Mode:** Resistance & Aerobic & Balance training + Tai-chi using Kinect systems  **Duration**: 60 min-session  **Frequency**: 3/week  **Length**: 3 months  **Dropout**: 13% | Multicomponent exercise program  **Duration**: 60 min-session  **Frequency**: 3/week  **Length**: 3 months  **Dropout**: 17% | **Tool**: CHS Frailty index  **Follow-up time**: 3 months |
| Yu, R. et al. (2019),  Hong Kong | 62.2 (±NA) | 134 pre-frail older adults  ≥ 50 years  (72/62) | Single-center, RCT, assessors-blinded  (Center-based) | **Mode:** Resistance & Aerobic training  **Duration**: 60 min-session  **Frequency**: 2/week  **Length**: 3 months  **Dropout**: 8% | Usual care  **Dropout**: 2% | **Tool**: CHS Frailty index  **Follow-up time**: 3 months |
| Barrachina-Igual, J. et al. (2020), Spain | 75.0 (±6.9) | 50 pre-frail older adults  ≥ 70 years  (27/23) | Single-center, RCT, assessors-blinded  (Center-based) | **Mode:** Balance & Flexibility & Aerobic training + Self-massage  **Duration**: 65 min-session  **Frequency**: 2/week  **Length**: 3 months  **Dropout**: 15% | Usual care  **Dropout**: 13% | **Tool**: CHS Frailty index  **Follow-up time**: 3 months |
| Seesen, M. et al. (2020), Thailand | 68.5 (±2.7) | 62 older adults  65-74 years  (30/32) | Single-center, non-RCT, assessors-blinded  (Center-based) | **Mode:** Strength & Balance training  **Duration**: 50 min-session  **Frequency**: 3/week  **Length**: 3 months  **Dropout**: 0% | Usual care  **Dropout**: 0% | **Tool**: CHS Frailty index  **Follow-up time**: 6 months |
| Sadjapong, U. et al. (2020), Thailand | 77.8 (±7.2) | 63 frail older adults  65-74 years  (31/32) | Single-center, RCT, assessors-blinded  (Center-based & Home-based) | **Mode:** Aerobic & Resistance & Balance training + Home-based exercise  **Duration**: 60 min-session  **Frequency**: 3/week  **Length**: 3 months + 3 months for home-based program  **Dropout**: 3% | Health education  **Dropout**: 0% | **Tool**: CHS Frailty index  **Follow-up time**: 6 months |
| Biesek, S. et al. (2021), Brazil | 71.2 (±4.5) | 36 pre-frail older women  ≥ 65 years  (18/18) | Single-center, RCT, assessors-blinded  (Center-based) | **Mode:** Resistance & neuromotor training with balance platform  **Duration**: 50 min-session  **Frequency**: 2/week  **Length**: 3 months  **Dropout**: 17% | Usual care  **Dropout**: 17% | **Tool**: CHS Frailty index  **Follow-up time**: 3 months |
| Merchant, R.A. et al. (2021), Singapore | NA | 197 pre-frail and frail older adults  ≥ 60 years  (122/75) | Multi-center, non RCT | **Mode:** Resistance & Balance training (Dual-task exercise by health coach)  **Duration**: 60 min-session  **Frequency**: 2/week  **Length**: 3 months  **Dropout**: 18% | Dual-task exercise by volunteer  **Dropout**: 49% | **Tool**: CHS Frailty index  **Follow-up time**: 6 months |
| Casas-Herrero, Á. et al. (2022), Spain | 84.1 (±4.8) | 188 older adults  > 75 years  (88/100) | Multi-center, RCT, assessors-blinded  (Home-base) | **Mode:** Resistance & Balance & Endurance training + gait training  **Duration**: 30 min-session  **Frequency**: 3/week + walking 5/week  **Length**: 3 months  **Dropout**: 48% | Usual care  **Dropout**: 28% | **Tool**: CHS Frailty index  **Follow-up time**: 3 months |
| Ng, T.P. et al. (2015), Singapore | 69.9 (±4.6) | 98 prefrail and  frail old adults  ≥ 65 years  (48/50) | RCT,  assessors-blinded  (Center-based & Home-based) | **Mode**: Strength & Balance training  **Duration**: 90 min-session  **Frequency**: 2/week + daily individual assigned (home-based)  **Length**: 3 months + 3 months for home-based program  **Dropout**: 0% | Usual care  **Dropout**: 6% | **Tool**: CHS Frailty index  **Follow-up time**: 12 months |
| Chan, D-C. et al. (2016), Taiwan | 71.6 (±4.3) | 289 old adults  65–79 years (143/146) | Multi-center, RCT,  assessors-blinded  (Center-based) | **Mode:** Strength & Balance & Flexibility training + Low-level care  **Duration**: 55-65 min-session  **Frequency**: 2/week  **Length**: 6 months  **Dropout**: 17% | Low-level care (health education & home exercise program)  **Dropout**: 19% | **Tool**: CHS Frailty index  **Follow-up time**: 12 months |
| Huguet, G. et al. (2018), Spain | 84.5 (±3.5) | 200 pre-frail old adults  ≥ 80 years  (100/100) | Multi-center, RCT,  assessors-blinded  (Center-based & home-based) | **Mode:** Strength & Balance & Flexibility & Aerobic training  **Duration**: 60 min-session & walking 30 min-session  **Frequency**:  Center-based: 9/fortnightly  Home-based: 3/week (walking)  **Length**: 6 months  **Dropout**: 15% | Usual care  **Dropout**: 12% | **Tool**: CHS Frailty index  **Follow-up time**: 12 months |
| Arrieta, H. et al. (2019), Spain | 84.9 (±6.9) | 112 older adults  ≥ 70 years  (57/55) | Multi-center, RCT, assessors-blinded  (Center-based) | **Mode:** Strength & Balance training  **Duration**: 60 min-session  **Frequency:** 2/week  **Length**: 6 months  **Dropout**: 25% | Low-intensity activities  **Dropout**: 18% | **Tool**: CHS Frailty index  **Follow-up time**: 12 months |
| Courel-Ibáñez, J. et al. (2021), Spain | 87.1 (±7.1) | 44 frail older adults  ≥ 75 years  (22/22) | Single-center, RCT, assessors-blinded  (Center-based) | **Mode**: Strength & Balance & Stretching training (Long training-short detraining: LT-SD)  **Duration**: 40-60 min-session  **Frequency**: 3/ week & walking 2/ week  - week 1-24: Long training  - week 25-30: Short detraining  **Length**: 6 months  **Dropout**: 9% | Center-based exercise program (Short training-long detraining: SD-LT)  - week 1-4: short training  - week 5-18: short detraining  **Dropout**: 0% | **Tool**: CHS Frailty index  **Follow-up time**:  7 months |
| Cameron, I.D. et al. (2013), Australia | 83.3 (±5.9) | 241 frail old adults  ≥ 70 years  (120/121) | Single-center, RCT, assessors-blinded  (Home-based) | **Mode**: Strength & Balance & Endurance training  **Duration**: tailored to individual’s physical impairments by the physiotherapists  **Frequency**: 3-5/week (10 sessions)  **Length**: 12 months  **Dropout**: 11% | Usual care  **Dropout**: 10% | **Tool**: CHS Frailty index  **Follow-up time**: 12 months |
| Cesari, M. et al. (2015), USA | 76.8 (±4.2) | 424 old adults  70–89 years with a sedentary lifestyle  (213/211) | Multi-center, RCT, assessors-blinded  (Center-based & Home-based) | **Mode**: Strength & Balance & Flexibility & Endurance training  **Duration**: 40-60 min-session  **Frequency**:  -week 1-8: Center-based 3/week  -week 9-24: Center-based 2/week + Home-based 3/week  -week 25-56: Home-based with option center-based 1-2/week  **Length**: 12 months  **Dropout**: NR | Health education  **Dropout**: NR | **Tool**: CHS Frailty index  **Follow-up time**: 12 months |
| Serra-Prat, M. et al. (2017), Spain | 78.3 (±4.5) | 172 pre-frail adults  ≥ 70 years  (80/92) | Multi-center, RCT,  assessors-blinded  (Home-based) | **Mode:** Aerobic & Strength & Balance training  **Duration**: 20-25 min-session &  30-45 min-session (walking)  **Frequency**: 4/week & walking 4/week  **Length**: 6 months  **Dropout**: 24% | Usual care  **Dropout**: 22% | **Tool**: CHS Frailty index  **Follow-up time**: 12 months |
| Liu, Z. et al. (2018), USA | 77.8 (±5.9) | 1,635 old adults  70-89 years  (818/817) | Multi-center, RCT, assessors-blinded  (Center-based & Home-based) | **Mode**: Strength & Balance & Flexibility training  **Duration**: 20 min-session & 30 min-session (walking)  **Frequency**:  2/week (Center-based) & 3-4/week (Home-based)  **Length**: 6 months  **Dropout**: 30% | Health education  **Dropout**: 28% | **Tool**: CHS Frailty index  **Follow-up time**: 12 months |
| García-Vigara, A., et al. (2024), Spain | 67.8 (±4.7) | 252 postmenopausal women  ≥ 60 years  (126/126) | Single-center, non-RCT, assessors-blinded  (Center-based) | **Mode**: Strength & Balance & Flexibility & Endurance training  **Duration**: 60 min-session  **Frequency**: 2/week (Center-based)  **Length**: 12 months (12 wk. professional supervised + 36 wk. group supervised)  **Dropout**: 0% (adherence <50% of session, n = 16/126 (12.70%)) | Usual activity  **Dropout:** 0% | **Tool**: CHS Frailty index  **Follow-up time**: 12 months |
| Carnavale, B. F., et al. (2024), Brazil | 74.8 (±6.4) | 32 pre-frail adults  ≥ 65 years  (20/20) | Single-center, RCT, assessors-blinded  (Center-based) | **Mode**: Strength & Balance & Flexibility training  **Duration**: 60 min-session  **Frequency**:  3/week (Center-based)  **Length**: 3 months  **Dropout**: 20% | Usual activity  **Dropout:** 45% | **Tool**: CHS Frailty index  **Follow-up time**: 4 months |

**Table S2.** The characteristics of the eligible nutritional intervention studies.

| **Author (year)** | **Mean age,**  **year (±SD)** | **Participants**  **(Intervention/**  **Control)** | **Study designs (Study base)** | **Intervention** | **Control** | **Outcome measures** |
| --- | --- | --- | --- | --- | --- | --- |
| Kim, H. et al. (2015), Japan | 81.0 (±2.7) | 64 frail older women  ≥ 75 years  (32/32) | RCT,  Double-blind  (Home-based) | **Type**: Macronutrient  **Detail**: Milk fat globule membrane (protein and fat pill) supplementation  **Dose**: 1 gm./day  **Frequency:** 7/week  **Length**: 3 months  **Dropout**: 9% | Placebo  **Dropout**: 0% | **Tool**: CHS Frailty index  **Follow-up time**:  4 months |
| Biesek, S. et al. (2021), Brazil | 71.2 (±4.5) | 36 pre-frail older women  ≥ 65 years  (18/18) | RCT,  assessors-blinded  (Home-based) | **Type**: Macronutrient  **Detail**: Whey protein isolate, CHO, Fat, mineral & multivitamin supplementation  supplementation  **Dose**: 42 gm./day  **Frequency:** 5/week  **Length**: 3 months  **Dropout**: 17% | Usual care  **Dropout**: 17% | **Tool**: CHS Frailty index  **Follow-up time**:  3 months |
| Na, W. et al.  (2021), Korea | 80.8 (±7.0) | 62 community-dwelling old adults  (31/31) | RCT,  Double-blind  (Home-based) | **Type**: Macronutrient and micronutrient  **Detail**: Oral nutritional supplement (CHO, Protein, Fat), multivitamin (A, D, E, Folate), mineral (Ca, Phosphorus, Zinc)  **Dose**: 200ml, 200 kcal/day/dose  **Frequency:** 7/week  **Length**: 3 months  **Dropout**: 9.70% | Placebo  **Dropout**: 19.4% | **Tool**: K-Frail  **Follow-up time**:  3 months |
| Ng, T.P. et al. (2015), Singapore | 69.9 (±4.6) | 99 prefrail and  frail old adults  ≥ 65 years  (49/50) | RCT,  assessors-blinded  (Home-based) | **Type**: Macronutrient and micronutrient  **Detail**: Energy (CHO, Protein, Fat, Fiber), mineral & multivitamin supplementation  **Dose**: Caloric intake by about 20% & 1/3 of the recommended daily allowances of vitamins and minerals & variability in individual energy requirements, maximal tolerable intake to gain 0.5 kg/week  **Frequency:** 7/week  **Length**: 6 months  **Dropout**: 10% | Usual care  **Dropout**: 6% | **Tool**: CHS Frailty index  **Follow-up time**: 12 months |
| Badrasawi, M. et al. (2016), Malaysia | 68.5 (±6.3) | 58 pre-frail old adults  ≥ 60 years  (29/29) | RCT,  Double-blind  (Home-based) | **Type**: Macronutrient  **Detail**: L-carnitine supplementation  **Dose**: 1.5 gm./day  **Frequency:** 7/week  **Length**: 10 weeks  **Dropout**: 10% | Placebo  **Dropout**: 18% | **Tool**: CHS Frailty index  **Follow-up time**: 10 weeks |
| Guerville, F. et al. (2019), France | 75.3 (±4.4) | 790 old adults  ≥ 70 years  (398/392) | RCT,  Double-blind  (Home-based) | **Type**: Micronutrient  **Detail**: Omega 3 polyunsaturated fatty acid  **Dose**: 800 mg./day  **Frequency:** 5/week  **Length**: 36 months  **Dropout**: 15% | Placebo  **Dropout**: 11% | **Tool**: CHS Frailty index  **Follow-up time**:  36 months |
| Seesen, M. et al. (2020), Thailand | 68.5 (±2.7) | 62 older adults  65-74 years  (30/32) | Single-center, non-RCT,  (Home-based) | **Type**: Micronutrient  **Detail**: Black rice germ and band powder (Energy + phenolic compound, total flavonoids & anthocyanin) supplementation  **Dose**: 10 gm./day  **Frequency:** 7/week  **Length**: 6 months  **Dropout**: 0% | None  **Dropout**: 0% | **Tool**: CHS Frailty index  **Follow-up time**:  6 months |

**Table S3.** Subgroup and sensitivity analyses of multicomponent exercise studies

| **Intervention** | **Condition for analysis** | | **Studies included** | **Fixed-effect meta-analysis** | | | | **Random-effect meta-analysis** | | | |
| --- | --- | --- | --- | --- | --- | --- | --- | --- | --- | --- | --- |
|  |  |  |  | **RR** | **95% CI** | ***p* value** | **I²** | **RR** | **95% CI** | ***p* value** | **I²** |
| Multicomponent exercises | All | | 21 | 0.59 | 0.50-0.70 | <0.001 | 9.0 | 0.59 | 0.50-0.71 | <0.001 | 4.4 |
|  |  | *Length of intervention* | | | | | | | | |  |
|  | ≤ 3 months | | 11 | 0.50 | 0.34-0.73 | 0.001 | 0.6 | 0.52 | 0.35-0.76 | <0.001 | 0.0 |
|  | 6 months | | 5 | 0.53 | 0.36-0.79 | 0.002 | 29.1 | 0.52 | 0.33-0.83 | 0.006 | 19.9 |
|  | ≥ 12 months | | 5 | 0.65 | 0.53-0.81 | <0.001 | 16.7 | 0.66 | 0.53-0.81 | <0.001 | 0.0 |
|  |  | *Study design* | | | | | | | | |  |
|  | Randomized-controlled trials | | 18 | 0.56 | 0.46-0.67 | <0.001 | 3.9 | 0.55 | 0.45-0.67 | <0.001 | 6.8 |
|  | Controlled Clinical trials | | 3 | 0.92 | 0.58-1.46 | 0.737 | 0.0 | 0.92 | 0.58-1.46 | 0.738 | 0.0 |
|  |  | *Study base* | | | | | | | | |  |
|  | Center-based | | 11 | 0.66 | 0.49-0.90 | 0.008 | 1.8 | 0.67 | 0.48-0.93 | 0.018 | 5.4 |
|  | Center-based & Home-based | | 6 | 0.57 | 0.45-0.73 | <0.001 | 46.4 | 0.50 | 0.34-0.75 | <0.001 | 39.5 |
|  | Home-based | | 4 | 0.56 | 0.38-0.81 | 0.002 | 0.0 | 0.56 | 0.38-0.82 | 0.003 | 0.0 |
|  |  | *Type of exercise* | | | | | | | | |  |
|  | Strength & Balance & Endurance training | | 7 | 0.46 | 0.32-0.65 | <0.001 | 19.5 | 0.44 | 0.28-0.69 | <0.001 | 19.3 |
|  | Strength & Balance & Flexibility & Endurance training | | 9 | 0.59 | 0.44-0.79 | <0.001 | 15.5 | 0.58 | 0.40-0.84 | 0.004 | 18.8 |
|  | Strength & Balance & Flexibility training | | 2 | 0.73 | 0.54-0.97 | 0.032 | 0.0 | 0.73 | 0.54-0.97 | 0.032 | 0.0 |
|  | Strength & Balance training | | 1 | 0.56 | 0.27-1.19 | 0.133 | - | 0.54 | 0.27-1.19 | 0.133 | - |
|  | Strength & Endurance training | | 2 | 0.58 | 0.29-1.17 | 0.124 | 0.0 | 0.58 | 0.29-1.18 | 0.134 | 0.0 |

**Table S4.** Subgroup and sensitivity analyses of nutritional studies

| **Intervention** | **Condition for analysis** | | **Studies included** | **Fixed-effect meta-analysis** | | | | **Random-effect meta-analysis** | | | |
| --- | --- | --- | --- | --- | --- | --- | --- | --- | --- | --- | --- |
|  |  |  |  | **RR** | **95% CI** | ***p* value** | **I²** | **RR** | **95% CI** | ***p* value** | **I²** |
| Nutritional intervention | All | | 7 | 0.64 | 0.45-0.91 | 0.012 | 10.3 | 0.61 | 0.37-0.99 | 0.049 | 28.3 |
|  |  | *Length of intervention* | | | | | | | | |  |
|  | ≤ 3 months | | 4 | 0.76 | 0.37-1.55 | 0.450 | 0.0 | 0.76 | 0.37-1.56 | 0.455 | 0.0 |
|  | ≥ 6 months | | 3 | 0.60 | 0.40-0.90 | 0.014 | 67.8 | 0.49 | 0.19-1.25 | 0.136 | 65.8 |
|  |  | *Study design* | | | | | | | | |  |
|  | Randomized-controlled trials | | 6 | 0.63 | 0.44-0.91 | 0.013 | 25.0 | 0.59 | 0.34-1.03 | 0.065 | 36.3 |
|  |  | *Type of nutrient* | | | | | | | | |  |
|  | Macronutrients | | 2 | 0.86 | 0.32-2.30 | 0.767 | 0.0 | 0.86 | 0.32-2.30 | 0.767 | 0.0 |
|  | Micronutrients | | 3 | 0.78 | 0.51-1.21 | 0.271 | 0.0 | 0.78 | 0.51-1.21 | 0.271 | 0.0 |
|  | Macronutrients and Micronutrients | | 2 | 0.27 | 0.12-0.61 | 0.015 | 18.6 | 0.28 | 0.11-0.72 | 0.008 | 18.6 |
|  | Macronutrients + Macronutrients and Micronutrients | | 4 | 0.43 | 0.23-0.79 | 0.006 | 32.0 | 0.46 | 0.19-1.11 | 0.085 | 39.7 |


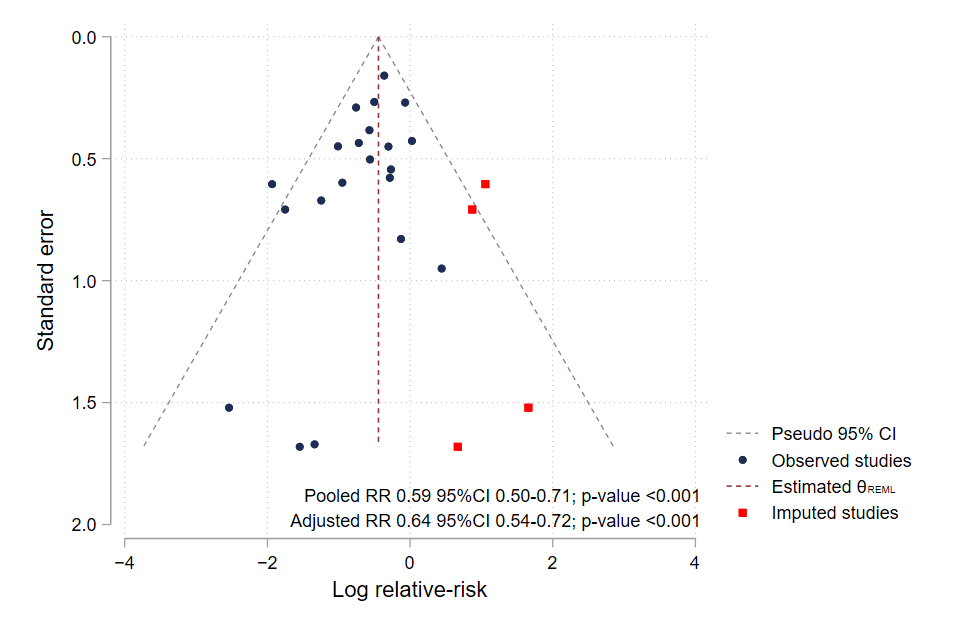
**Figure S1.** Funnel plot of the multicomponent excercises studies with trim and fill method
