# Supplementary figures and images for: Impact of multicomponent exercise and nutritional supplement interventions for improving physical frailty in community-dwelling older adults: a systematic review and meta-analysis
Source: BMC Geriatr. 2024 Nov 18;24:958. doi: 10.1186/s12877-024-05551-8 (PMC11571505; doi:10.1186/s12877-024-05551-8)

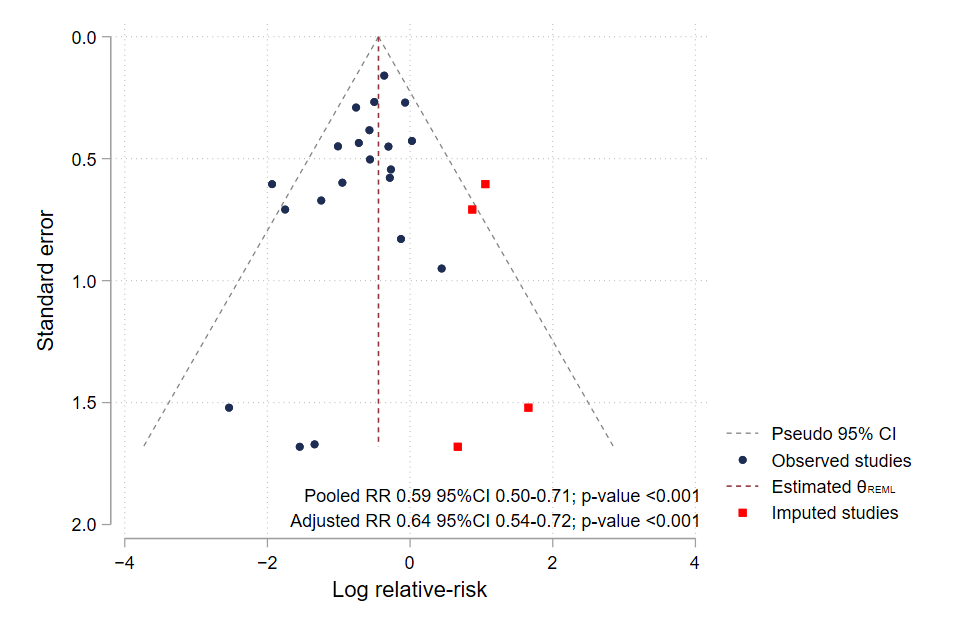

Supplement: Supplementary file 3 — Supplementary Material 3. [file 12877_2024_5551_MOESM3_ESM.tif]
